# Breast imaging findings in dermatofibrosarcoma protuberans: Case report and literature review

**DOI:** 10.1097/MD.0000000000042247

**Published:** 2025-04-25

**Authors:** Jie He, Zhizhi Tao, Wangwang Liu, Feifei Lou, Hongjie Hu

**Affiliations:** a Department of Radiology, Sir Run Run Shaw Hospital, Zhejiang University School of Medicine, Hangzhou, China; b Department of Pathology, Sir Run Run Shaw Hospital, Zhejiang University School of Medicine, Hangzhou, China.

**Keywords:** breast, dermatofibrosarcoma protuberans, diagnosis, imaging

## Abstract

**Rationale:**

Dermatofibrosarcoma protuberans (DFSP) of the breast is a rare condition and is often misdiagnosed as benign breast lesions, leading to delays patient treatment. Complete surgical resection with negative margins is the primary treatment option. In this study, we report a case of a patient with DFSP, with clinical and imaging presentations and treatment modalities.

**Patient concerns:**

A 15-year-old girl was admitted with a gradually enlarging right breast mass over 3 years. Physical examination revealed symmetrical breasts with no deviation or depression of the nipples, discharge, or orange peel texture or dimpling. A palpable mass of approximately 30 × 25 mm was located in the 2 o’clock position of the right breast.

**Diagnoses:**

The imaging characteristics of the breast mass were similar to those of breast fibroadenoma. A preoperative radiological diagnosis of breast fibroadenoma was made; however, postoperative pathology confirmed DFSP.

**Interventions:**

The patient underwent local resection of the right breast mass under general anesthesia. The diagnosis of a malignant tumor with capsular invasion of the right breast prompted a second surgery 16 days later that involved radical extended resection.

**Outcome:**

The patient has been followed up regularly for approximately 2 years with no signs of recurrence.

**Lessons:**

Breast DFSP is frequently misdiagnosed as a benign lesion owing to its imaging characteristics and symptom presentation, risking treatment delays. Surgical resection is the standard treatment; however, nonsurgical treatment is also effective. This study highlights the importance the diagnostic accuracy and treatment of this condition.

## 1. Introduction

Dermatofibrosarcoma protuberans (DFSP) constitutes a rare, slow-growing category of soft-tissue tumors, representing approximately 1% of all soft-tissue sarcomas, with an estimated annual incidence of about 4 cases per 100,000 individuals.^[1,2]^ Although DFSP is a malignant tumor, it rarely metastasizes and has a good prognosis, with a 10-year survival rate exceeding 90%. The tumor has been reported in patients of all ages, with a more common age of onset between 20 and 50 years, with some studies indicating a higher prevalence among African Americans.^[3]^ Clinically, DFSP typically presents as a flesh-colored, red papule, or nodule with a firm texture.^[4]^ While DFSP can develop on any part of the body, it is most commonly observed on the trunk and limbs, followed by the head and neck.^[5]^ DFSP is slow-growing, often not noticeable when it first appears, and often grows to a relatively large size before being detected.

DFSP in the breast is rare and has been reported as an isolated case in the literature.^[6–12]^ The patients are often misdiagnosed with benign breast lesions, such as fibroadenomas, owing to tumor’s indolent growth and nonspecific imaging features.^[13]^ Patients with breast DFSP who detect a mass are typically first evaluated using a mammogram or ultrasound. When DFSP is diagnosed via biopsy, contrast-enhanced magnetic resonance imaging (MRI) of the breast may be used to determine the extent of the tumor.^[14,15]^ Complete resection with negative margins remains the primary treatment approach, as incomplete excision is associated with higher recurrence rates.^[16]^ Regular postoperative imaging is recommended to monitor for local recurrence.

Here, we report the clinical presentation, imaging findings, and treatment of a patient with DFSP.

## 2. Methods

### 2.1. Ultrasound examination

Whole-breast handheld ultrasound (US) was conducted using a 5 to 12 MHz linear array transducer (iu22 machine, Philips, Bothell). This procedure was performed by a sonographer specializing in breast imaging with 10 years of experience. Routine scanning of the breast US and axillary and internal mammary lymph nodes was performed while the patients were in a supine position.

### 2.2. MRI examination

Breast MRI was performed using a 1.5 T scanner (Signa HD excite; GE Healthcare, Milwaukee). Before the contrast medium was administered, axial T2-weighted and axial diffusion-weighted images (2 b-values of 0 and 800 s/mm^2^) were acquired. Pre-scan of fat-suppressed T1-weighted imaging was performed before scanning fat-suppressed multi-phase dynamic enhanced T1WI, and images were obtained in 7 phases at intervals of 70 seconds, following the injection of a gadolinium-based contrast agent. A gadolinium-based agent (Gd-DTPA; Beijing Beilu Pharmaceutical Co., Ltd., Beijing, China) was injected using a high-pressure syringe at a rate of 3 mL/s and dose of 0.1 mmoL/kg body weight, followed by a 20 mL saline flush at the same flow rate.

## 3. Case presentation

A 15-year-old girl presented with a mass in her right breast for 3 years and was admitted to the Department of Surgery Oncology of Sir Run Run Shaw Hospital of Zhejiang University School of Medicine on July 12, 2022. The patient found a right breast mass that was identified by chance after an impact in the anterior chest area 3 years prior, with no tenderness, nipple bleeding, or discharge; and visited the local hospital on her own at that time and was advised to follow-up regularly. Over the past 3 years, the patient reported that the mass had gradually increased in size. The patient had not undergone any prior breast surgery due to trauma, and with no familial occurrence of malignant tumors. The patient’s respiratory rate was recorded at 18 breaths per min, accompanied by a body temperature of 37.2°C, a pulse rate of 71 beats per minute, and a blood pressure reading of 106/61 mm Hg. On physical examination, both breasts appeared symmetrical, showing no deviation or depression of the nipples, no bleeding or discharge from the nipples, and an absence of an orange peel appearance or signs of dimpling. A palpable mass of approximately 40 × 25 mm in size was identified in the 2 o’clock direction of the right breast, with clear boundaries, movable scar-like nodules visible on the surface, slightly red and swollen locally, with no ulceration. No obvious mass was detected in the left breast, and no enlarged lymph nodes were observed in the bilateral supra- and subclavicular regions, or axillary regions.

**Figure 1.**
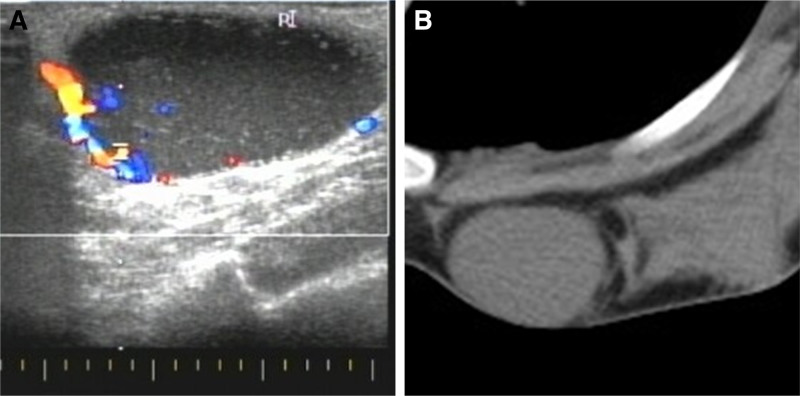
A 15-yr-old woman with a lesion in her right breast that was diagnosed as fibroadenoma. (A) Ultrasonographic images reveal a 43 × 21 mm hypoechoic mass in the lower inner quadrant of the right breast that has clear boundaries, regular morphology, peripheral visible capsule, internal visible striated structure, and slightly abundant blood flow signal in the peripheral area. (B) Computed tomography images of the chest present an iso-density mass in the lower inner quadrant of the right breast, approximately 39 × 29 mm in size.

**Figure 2.**
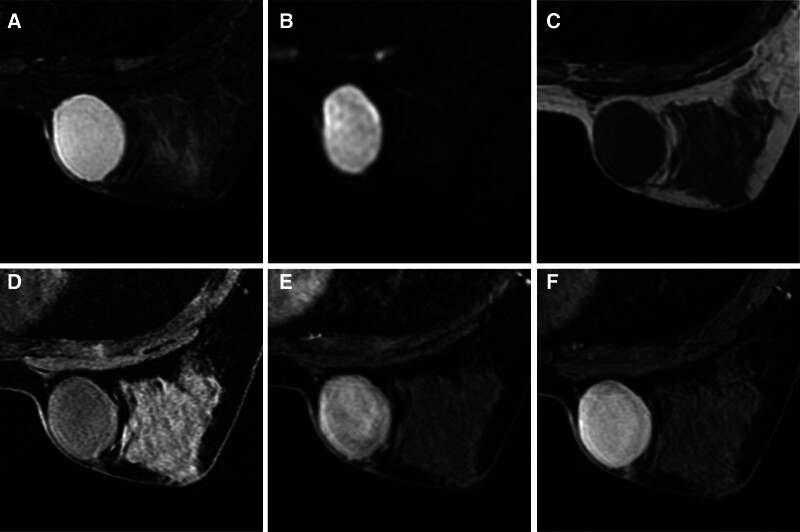
Axial fat-saturated, T2-weighted image (A) and diffusion-weighted image (B) reveal hypointensity oval mass. In axial, nonfat-saturated, T1-weighted images (C) and pre-scan, fat- saturated, T1-weighted images (D), the mass exhibits slightly hypointense when compared to the surrounding breast fibroglandular tissue. After contrast medium administration, the mass shows defined, fast enhancement in the initial phase (E) and persistent in the late phase (F).

Breast US (Fig. 1A) revealed a hypoechoic mass approximately 43 × 21 mm in size in the lower inner quadrant of the right breast, with clear boundaries, regular morphology, a peripheral visible capsule, an internal visible striated structure, and a slightly abundant blood flow signal in the peripheral area. The blood flow resistance index was 0.475. No enlarged lymph nodes was observed in the bilateral axillae or supraclavicular regions. Breast fibroadenoma was diagnosed using ultrasonography. Thoracic computed tomography (Fig. 1B) revealed an iso-dense mass in the lower inner quadrant of the right breast that measured approximately 39 × 29 mm, without lobulation or burrs. The MRI results are shown in Figure 2A to F. The oval mass was hyperintense on T2-weighted images and diffusion-weighted images and slightly hypointense on nonfat-suppressed and fat-suppressed T1-weighted images. After contrast agent injection, the mass showed defined, fast enhancement in the initial phase and persistence in the delayed phase. We made an MRI diagnosis of a fibroadenoma of the breast.

**Figure 3.**
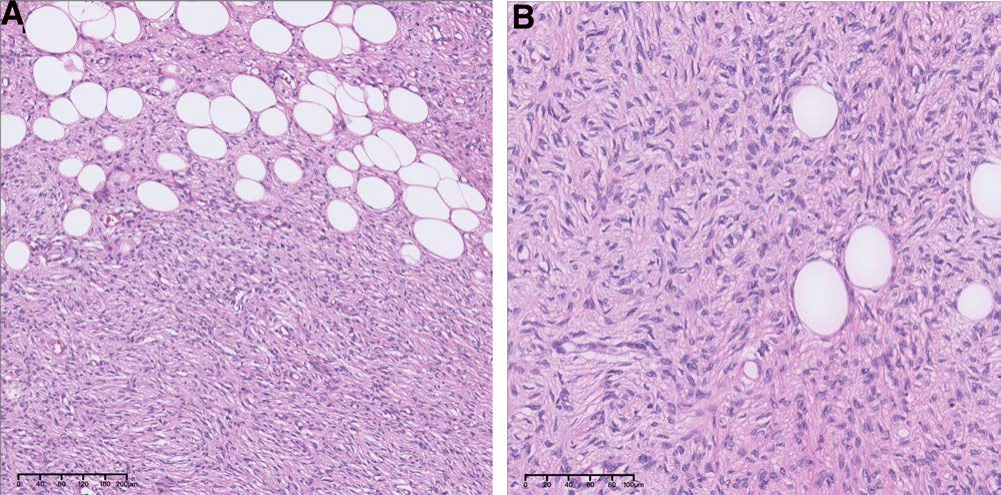
Histopathological examination excision of the lesion. The neoplastic cells invade the subcutaneous adipose tissue, leading to a characteristic honeycomb appearance. This tissue is composed of cytologically consistent spindled tumor cells, organized in storiform, whorled, or cartwheel patterns of growth. There is minimal cytological atypia, and the mitotic activity was low. The collagenous stroma features small blood vessels. (A) hematoxylin and eosin staining, ×10 and (B) hematoxylin and eosin staining, ×20.

**Figure 4.**
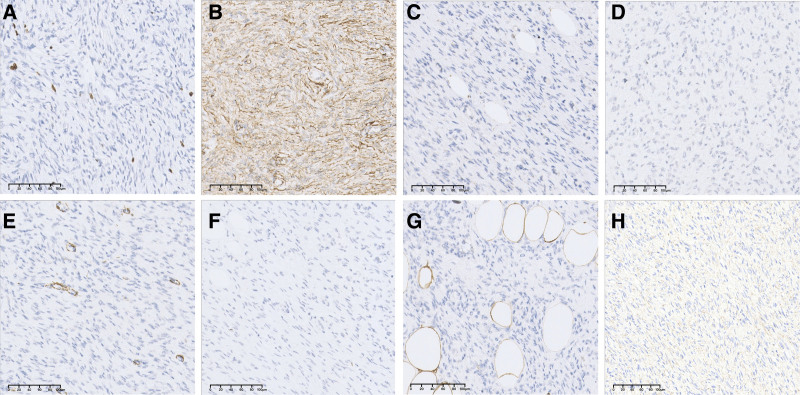
Ki-67 positivity is identified in < 5% of tumor cells (×20) (A). Immunohistochemical findings reveal positive staining for CD34 (×20) (B), and negative for STAT6 (×20) (C), epithelial membrane antigen (×20) (D), smooth muscle actin (×20) (E), desmin (×20) (F), S-100 protein (×20) (G), and beta-catenin (×20) (H).

Considering the aforementioned clinical symptoms and imaging results, the first consideration was a benign breast tumor with a high possibility of fibroadenoma. Because the mass was large and had surgical indications, a decision was made following discussions with the patient and her family to proceed with the surgical removal of the localized mass in the right breast. On July 13, 2022, a local resection of the right breast mass was performed under general anesthesia. Intraoperatively, a 40 × 30 mm mass was observed at the edge of the gland in the lower inner quadrant of the right breast, which was tough, with clear borders and an intact capsule, and was completely excised together with the capsule. The dissected mass was yellowish in color and solid. Histopathological examination revealed that the tumor exhibited a widespread infiltration of both the dermis and subcutis. Neoplastic cells invaded the subcutaneous adipose tissue, producing a characteristic honeycomb pattern. The tumor consisted of cytologically consistent spindled tumor cells organized in storiform, whorled, or cartwheel growth configurations. Minimal cytological atypia was observed, and the mitotic activity remained low. The collagen-rich stroma encompassed the small blood vessels (Fig. 3). No evidence of necrosis or atypical mitotic activity was observed, and the Ki-67 index was <5% (Fig. 4A). Immunohistochemical analysis revealed that the tumor cells expressed CD34, but were negative for STAT6, epithelial membrane antigen, smooth muscle actin, desmin, S-100 protein, and beta-catenin (Figs. 4B–H). Postoperative pathology revealed right breast dermatofibrosarcoma protuberans with tumor invasion into the capsule. The diagnosis of a malignant tumor with capsular invasion of the right breast prompted a second radical extended resection surgery 16 days later. The postoperative margins were negative, and the patient has been undergoing regular postoperative reexaminations and for almost 2 years with no recurrence.

## 4. Discussion

Dermatofibrosarcoma protuberans is a rare soft-tissue sarcoma with a low likelihood of distant metastasis.^[2]^ Clinically, DFSP often presents as small, firm, painless, flesh-colored or reddish plaques that gradually increase in size, sometimes forming larger masses. The tumor may affect only the top layer of the skin, and sometimes penetrate deep into the subcutaneous tissue and even the muscles.^[17]^ As DFSP typically does not cause pain in its early stages, patients often fail to notice it, resulting in delays of months or even years.^[18]^ Additionally, clinicians also tend to mistake DFSP for other tumors.^[19]^ Prior studies have shown that it can take a long time, sometimes up to several years, from initial symptoms to the final diagnosis. In our patient’s case, a right breast mass was discovered incidentally, following trauma to the anterior chest area 3 years prior, but it was not brought to medical attention until the tumor had grown significantly. Early diagnosis and surgical excision are crucial to prevent the tumor from transforming into a malignant form, such as fibrosarcoma.^[7]^ The prevalence of DFSP in the breast is low, and its radiological features are nonspecific. Therefore, clinicians may find it challenging to confirm a diagnosis based solely on imaging.^[13,15]^

Common imaging modalities for breast diseases include mammography, ultrasonography, and MRI. On mammography, DFSP tends to appear as an exophytic growth with a slightly lobulated mass and well-defined noncalcified margins. On ultrasound, the tumor presented as a well-defined mass that is lobulated in some regions, most of which were hypoechoic, while some were mixed hypoechoic or hyperechoic. The intensity of the echoes in our patient was consistent with the findings in most patients. Color Doppler imaging revealed moderate and peripheral vascularization, and on MRI, the mass tends to appear as a well-defined lesion close to the skin, with a low or equal signal on T1-weighted images, high signal on T2-weighted images, and e rapid and marked enhancement after contrast medium injection.^[13]^ Our patient showed persistent enhancement in the enhancement-delayed phase, in addition to the above findings on MRI.

Dermatofibrosarcoma protuberans is challenging to diagnose and is often misdiagnosed owing to similarities in appearance to other conditions. Common misdiagnoses include vascular lesions, benign proliferative lesions, dermatofibromas, birthmarks, giant cell fibroblastomas, and atrophy-based skin conditions.^[20]^ DFSP is difficult to diagnose accurately using breast imaging and is easily misdiagnosed as a benign tumor. Imaging techniques, especially MRI, can help accurately assess tumor boundaries and guide the development of precise surgical treatment plans, owing to their exceptionally high soft-tissue contrast resolution. However, whether MRI can reduce positive margins and improve outcomes of breast-conserving surgery remains controversial.^[21,22]^ Confirmation of the diagnosis of DFSP usually relies on a biopsy by puncture or excision. Regarding gross pathology, the tumor has no capsule, the boundary are not clear, and the cut surface is gray-white or gray-red if there is hemorrhage, solid, nodular or lobular, tough, or medium texture, with a maximum diameter of 1 to 15 cm. Microscopic analyses have revealed spindle cell tumors arranged in storiform, whorled, or cartwheel growth patterns. Blood vessels were observed at the center of the mat-pattern structure. The cells were high in density with mild/moderate dysplasia, had no tumor giant cells or multinucleated giant cells, and had a variable number of nuclear divisions. Portions of the tumor resembled fibrosarcoma-like transformation with increased nuclear division. In general, coagulative necrosis did not occur. The tumor showed infiltrative growth along the fat lobules and intervals and invades the fat. Immunohistochemical results often contribute to the diagnosis of DFSP by showing CD34 positivity, which is consistent with our findings.

Achieving complete resection with tumor-free margins is the standard and mainstay of treatment for DFSP.^[23–25]^ However, considerable controversy exists within the academic community regarding the choice between wide local excision and Mohs micrographic surgery (MMS) as preferred treatment approach. Numerous retrospective studies have compared these 2 techniques, but the lack of clinical trials directly evaluating them, combined with selective bias reported in the literature, complicates accurate interpretation of the findings.^[16]^ However, MMS, a treatment preferred by many patients owing to its tissue-sparing benefits and higher cure rates compared to those with other modalities, has been recommended by the National Comprehensive Cancer Network guidelines.^[16,20]^ In our case, the patient underwent radical surgery, achieving negative surgical margins. As a result, a better outcome was obtained, and the patient has been followed up for almost 2 years without recurrence. Failure to completely remove the tumor during surgery, particularly when cancer cells are detected at or near the resection margin, is a key factor contributing to poor prognosis. In such cases, surgery can be repeated with an extended resection or administering radiotherapy may be necessary.^[7]^ In cases where surgical resection is not feasible, such as in cases of unfavorable tumor location or patient refusal to undergo surgery, nonsurgical treatment options, such as local radiation therapy or targeted chemotherapy, may be considered. Additionally, targeted chemotherapeutic agents such as imatinib, a tyrosine kinase inhibitor that is effective against tumors expressing the platelet-derived growth factor-B receptor,^[26]^ can be used as neoadjuvant chemotherapy, for preoperative reduction of tumor size, and for improved surgical outcomes. In addition to medical and surgical treatments, the psychological and developmental effects of DFSP should not be ignored.^[20,27]^ The surgical removal of large or conspicuous lesions significantly affects the physical appearance and emotional well-being of young patients.^[20,23]^ Consequently, it is essential to provide psychosocial support to patients and their families, as this support helps reduce anxiety and promotes an optimistic perspective on treatment and recovery. Finally, considering the high risk of DFSP recurrence, long-term follow-up is crucial to ensure that patients do not experience local recurrences, which may occur even after complete excision. Regular clinical evaluations are vital for monitoring the treatment area and allowing swift action in patients with recurrences.^[16,20,28]^

## 5. Conclusion

The breast is an uncommon site of DFSP, and it is often misdiagnosed as a benign tumor based on preoperative imaging and clinical symptoms, potentially causing delays in treatment. Currently, the primary approach for managing DFSP involves surgical resection. In cases where surgery resection is not feasible, nonsurgical treatment options 1may be a viable option. Our findings might help clinicians diagnose and treat such diseases more accurately.

## Acknowledgments

We would like to thank Editage (www.editage.cn) for English language editing.

## Author contributions

**Data curation:** Zhizhi Tao.

**Methodology:** Wangwang Liu, Feifei Lou, Hongjie Hu.

**Writing – original draft:** Jie He, Wangwang Liu.

**Writing – review & editing:** Feifei Lou, Hongjie Hu.
